# Habitat Selection by Free-Grazing Sheep in a Mountain Pasture

**DOI:** 10.3390/ani14131871

**Published:** 2024-06-25

**Authors:** Virginia Larraz, Olivia Barrantes, Ramón Reiné

**Affiliations:** 1 Departamento de Ciencias Agrarias y del Medio Natural, Universidad de Zaragoza, 50009 Zaragoza, Spain; olivia.barrantes@unizar.es (O.B.); rreine@unizar.es (R.R.); 2 Instituto Agroalimentario de Aragón-IA2, Universidad de Zaragoza-Centro de Investigación y Tecnología Agroalimentaria de Aragón, 50013 Zaragoza, Spain

**Keywords:** grassland conservation, free-roaming sheep, GPS collars, grazing preferences, use of territory

## Abstract

**Simple Summary:**

In the Pyrenees, traditional guided herding practices have often shifted to free-ranging flocks, impacting the management of mountain grasslands. This study utilizes GPS-based livestock tracking systems and Geographic Information Systems (GISs) to understand livestock behavior in remote areas. Tracking a flock of free-ranging sheep during the summers of 2019 to 2021, preferences for grazing on specific geomorphological features and plant communities were assessed. The results indicate that sheep prefer altitudes between 2400 and 2600 m and SE-, SW-, and E-facing sites with slopes under 20%. Preferred pastures include *Festucion eskiae*, *Primulion intricatae*, and *Nardion strictae*, while *Festucion scopariae* communities are rejected. This knowledge contributes to the efficient management of summer mountain pastures and aids in their preservation.

**Abstract:**

For centuries, mountain grasslands have been part of the grazing cycle of sheep and cattle in extensive management systems in the Pyrenees; however, traditional guided herding has been, in many cases, replaced by free-ranging flocks at these pastures. The goal of this study was to analyze the grazing behavior of free-ranging sheep in mountain pastures using GPS-based tracking systems and Geographic Information Systems. During the summer seasons of 2019, 2020, and 2021, a transterminant flock of free-ranging sheep was tracked with GPS devices attached to collars at the mountain pastures of Collarada mountain (Central Pyrenees, Spain). Preferences for grazing on certain geomorphological features (slope, aspect, and altitude) and different plant communities present in the area were evaluated using the Manly’s standardized preference index. The results show that sheep prefer altitudes between 2400 and 2600 m; SE-, SW-, and E-facing sites; and slopes under 20%. The preferred pastures were *Festucion eskiae*, *Primulion intricatae*, and *Nardion strictae*; however, they reject *Festucion scopariae* communities. This study demonstrates the effectiveness of GPS and GIS technologies in monitoring free-ranging sheep activity, providing valuable data for enhancing pastoral practices and ensuring sustainable pasture use.

## 1. Introduction

Mountain pastures are situated across the montane, subalpine, and alpine altitudinal zones of the Pyrenees, where they reach the highest elevations of the mountain range. Typically, they consist of open and expansive spaces located above the uppermost forest cover and are often subjected to various degrees of human alteration. These extensive pastoral landscapes are generally communally owned and have hosted, for centuries, transhumant or local sheep, along with larger livestock [1]. In addition to high-quality forage for traditional livestock grazing, mountain pastures provide a wide range of other ecosystem functions and services for society [2,3].

Historically, mountain agroecosystems remained relatively stable, primarily due to the relative isolation of this socio-economic system [4]. The most significant changes have occurred quite recently, since the mid-20th century, and have accelerated in recent decades. These shifts include abandonment of agro-pastoral activities, land-use changes, and a decrease in the number of farms that own larger flocks [4,5]. Due to the decline in livestock numbers and a shortage of shepherds, significant changes have also occurred in the management of animals in the mountain pastures in recent years. From a system of guided herding of flocks, there has been, in many cases, a transition to livestock that roam freely in the mountain pastures, resulting in an uneven spatial distribution of grazing animals across pastures [6]. In this way, selective foraging patterns can result in overgrazing of preferred areas while other areas receive little grazing attention [7]. The use of new technologies, specifically GPS-based livestock tracking systems, combined with Geographic Information Systems (GISs), presents an opportunity to facilitate the shepherd’s remote control of flock movements.

Sheep play an important role in the preservation of highland summer pastures due to their foraging behavior, which is known to have a discernible impact on plant diversity and structural attributes. The combined influence of trampling, defoliation, seed dispersal, and nutrient redistribution through fecal deposits can modulate vegetation cover, structure, and botanical composition [8].

Drivers of sheep movement across the landscape include abiotic, biotic, and social factors [9]. Sheep’s use of mountain slopes was found to be affected by several factors related to the mountainous terrain [10]. Vegetation type, phenology, and abundance are driving biotic factors behind large-scale foraging decisions [11], as the location of preferred vegetation directly impacts sheep grazing site selection [12].

Habitat selection by wild animals has been extensively explored for numerous species of ecological importance, compared with fewer studies of habitat selection by domestic grazing livestock [13]. New technologies (e.g., GPS collars) and advances in remote sensing have made it possible to collect animal location data on unprecedented spatial and temporal scales [14]. This, in turn, has fueled the development of new methods for modeling animal movement and for linking the locations of individual animals to important features of their environment (i.e., resources and environmental conditions) [15]. Information from the GIS on habitat characteristics, such as topography and vegetation, is often utilized in the development of variables for modeling resource selection [16]. Rivero et al. [17] reviewed 84 studies across diverse production systems all over the world regarding the site use preference of grazing cattle through GPS tracking. The results showed that some variables, such as stocking rate, water and shade location, weather conditions, terrain, and vegetation characteristics, have a significant impact on the behavior of grazing cattle. Spatial landscape use has been studied more in cattle than in small ruminants [9]. For instance, Putfarken et al. [18] studied the site use of grazing cattle and sheep in a large-scale pasture landscape typical of the lowlands of northern central Europe, combining GPS/GIS, and found that sheep preferred dry and nutrient-poor habitats and grazing sites close to their shed. Pittarello et al. [19] studied plant species selection by sheep in semi-natural dry grasslands in the Italian Alps, and their results suggest that sheep under low-intensity grazing conditions exert a specific plant species selection in abandoned dry grasslands. Ormaechea [20] studied the spatial distribution patterns of sheep in extensive livestock systems in Southern Patagonia and found that the distribution of sheep is primarily influenced by the interaction between terrain elevation, forage availability, and proximity to fences. Baum [21] used spatial locations of domestic sheep obtained from GPS collars to study habitat selection on a summer mountain range in Utah (United States) and observed that sheep selected gentle terrain, areas close to water, northern-facing slopes, and higher elevations but avoided slopes. Plaza et al. [22] used satellite-based tracking and remote-sensing technologies to monitor the spatial behavior of grazing sheep in a Spanish ‘dehesa’ ecosystem, determining that flocks showed strong preferences for grazing areas with gentle north-facing slopes, where the herbaceous layer formed by pasture predominates.

The development of sustainable grazing management strategies can be enhanced by understanding the mechanisms behind observed landscape distribution and grazing patterns [9]. The selection of grazing sites by animals responds to a complex interaction of factors that must be studied at different spatio-temporal scales and according to the particularities of each location [10,11]. When a herbivore selects a particular type of forage, it is simultaneously choosing a type of habitat where the effort and energy cost of searching for that feed are minimized [23].

The purpose of this study was to evaluate the preferences of a free-ranging flock of sheep, tracked with GPS devices attached to collars, for grazing on several geomorphological features (slope, aspect, and altitude) and different vegetation types in a mountain summer pasture in the Central Pyrenees. The final result of the data being studied aims to provide insights into sheep grazing behavior and habitat selection, which are crucial for optimizing grazing management strategies and ensuring the sustainable use and preservation of mountain pastures.

## 2. Materials and Methods

### 2.1. Study Area

**Figure 1:**
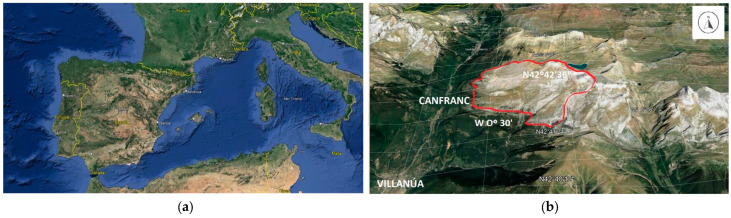
(**a**) Location map; (**b**) Collarada mountain (Villanua, Spain) and study area (red line). Source: Google Earth.

This study was conducted on the mountain grasslands of Collarada in the Central Pyrenees (Huesca, Spain) (Figure 1), which have an extension of 2100 ha. It is a karst landscape characterized by rugged and pronounced features, such as the Collarada Peak, which stands as its highest point at 2886 m, with a minimum elevation of 1100 m. The annual average temperature and cumulative rainfall in the period 1991–2021 were 7.4 °C and 1539 mm, respectively [24]. This area represents a typical highland summer pasture ecosystem in the Pyrenees, and it was chosen for its logistical feasibility.

Collarada mountain grasslands belong to the site ES2410023 “Collarada y Canal de Ip” of the Natura 2000 Network, protected under Habitat Directive 92/43/EEC (European Community). The lower third of the area is covered by *Pinus sylvestris* forest. The vegetation of the upper part is composed of herbaceous communities belonging to the phytosociological alliances *Saponarion caespitosae*, *Festucion scopariae*, *Bromion erecti*, *Primulion intrincatae*, *Nardion stricatae*, and *Festucion eskiae* [25].

The investigation was carried out on an area of about 850 ha (Figure 1b), where sheep ranged freely during the summer grazing seasons of 2019, 2020, and 2021.

### 2.2. Flock Monitoring

A flock of around 1000 sheep, belonging to 4 farmers of nearby villages (within 50 km range), grazes these summer pastures every year. Animals belong to ‘Rasa Aragonesa’, a local breed raised for meat production with a high degree of ruggedness, gregarious instinct, and pasturing ability that are well-adapted to the harsh environment of mountainous areas [26].

**Figure 2:**
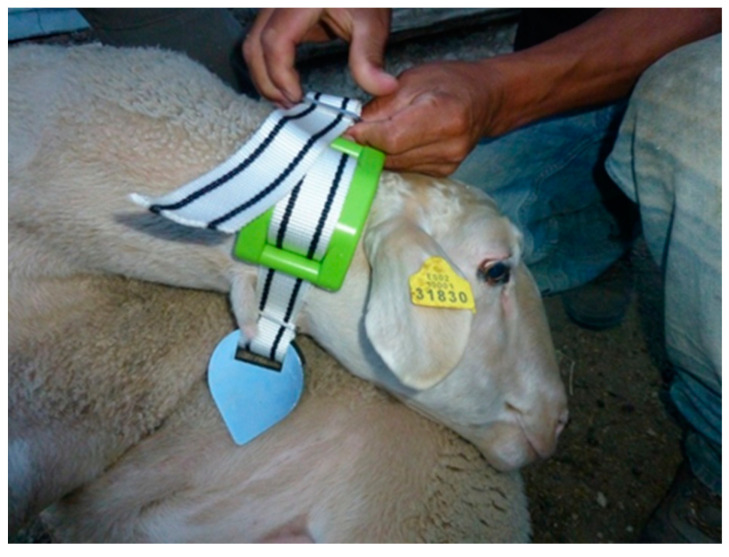
Sheep equipped with GPS device attached to collar.

Four ewes were randomly selected among the most robust sheep from the flock and equipped with GPS devices attached to collars (*Digitanimal SL*., Madrid, Spain) [27] (Figure 2). Since sheep are a species characterized by highly cohesive grazing behavior, we assumed the 4 selected animals were representative of the entire flock. The sampled animals were different every year, and sheep wore the collars continuously for the whole duration of the experiment.

The GPS collars (Figure 2) collected location data and temperature on the surface of the sheep at 11 min intervals to ensure sufficient battery life for the monitoring period. Devices transmitted the information to *Digitanimal* servers through an existing IoT network (SigFox) [28], which had good coverage within the study area. At the end of each grazing season, a *.csv* file with all positional and temperature data was provided by *Digitanimal*.

### 2.3. Geographical and Vegetation Preference Analyses

Geographical analyses were conducted using QGIS version 3.28 “Firenze” [29]. The altitude, aspect, and slope of the study area were obtained from the Digital Terrain Model (Instituto Geográfico Nacional of Spain, [30]). Aspect was then reclassified for analysis as a categorical variable with eight levels, corresponding to the cardinal directions (N, NE, E, SE, S, SW, W, and NW). The altitude of the study area, ranging from <2000 to >2600 m, was classified into 5 categories of 200 m intervals each. Slope was classified into 6 different categories (<10, 10–20, 20–30, 30–40, 40–50, and >50%). The vegetation map of Collarada was obtained from the Pyrenean Institute of Ecology, Jaca (Spain) [Gartzia and Gómez, unedited], and was used in [31]. Nine different vegetal communities were present in the study area. The area (ha) for each of these categories of resource units was calculated.

Data from the GPS device with the highest number of records were used for the calculations. A period of 65 days, from mid-July to mid-September, was considered for each year. The .csv file with all GPS positions, 15,582 in total, was incorporated into the QGIS project to obtain the altitude, aspect, slope, and vegetation type of each GPS position. Subsequently, the data were analyzed using Excel. The GPS locations were classified into 1 h range categories. Locations from 2 to 4 a.m. were used to determine the nighttime resting sites. Locations from 7 to 11 a.m. and from 3 to 7 p.m. were designated as “grazing”. This period, when the main activity of the flock is grazing, was determined by visual observation of sheep behavior in the field.

In order to evaluate sheep resource selection patterns across the landscape, we compared the availability of resources with their utilization during the grazing period. We calculated the Manly’s standardized preference index (B_i_) [13] for the aspect, altitude, slope, and vegetation categories. The index is based on the selection ratio W_i_, which is the proportional use (number of GPS positions found in each resource type) divided by the proportional availability (area) of each resource ‘i’ in the study area. A W_i_ value greater than 1 indicates a positive selection for the resource, and a value below 1 indicates avoidance of the resource. A value around 1 indicates that the resource was used proportionally to its availability, and no resource selection was noted. Standardized indexes are an alternative way to present the preference indexes so that they add to 1. Therefore, standardized indexes express the estimated probability of selection of a particular resource if all resources were equally frequent. We used function ‘widesI’ in Rpackage ‘adehabitatHS’ [32] to explore resource selection by sheep, which may be used when designs I [13] are involved (habitat use and availability are measured at the population level, animals are not identified, and the resource units are assumed to be sampled for the entire study area). The function tests whether all the available resources are used randomly with the log-likelihood Chi2 [13]. Manly’s selectivity measure (selection ratio = used/available) is computed, the preference/avoidance is tested for each resource type, and the differences between selection ratios are computed and tested. Significant resource selection is inferred after Bonferroni correction for multiple test (Bonferroni-corrected *p*-value = α/*n*, where α = 0.05 and *n* = number of tests performed).

We used the Friedman test to see whether sheep choose resources similarly each year (2019, 2020, 2021). Differences in selection between the different months (July, August, and September) and between the morning (7–11 h) and afternoon (15–17 h) grazing periods were also analyzed with the Friedman test, which was also used to compare the mean temperature over the study period for the 3 years (2019, 2020, 2021).

## 3. Results

**Figure 3:**
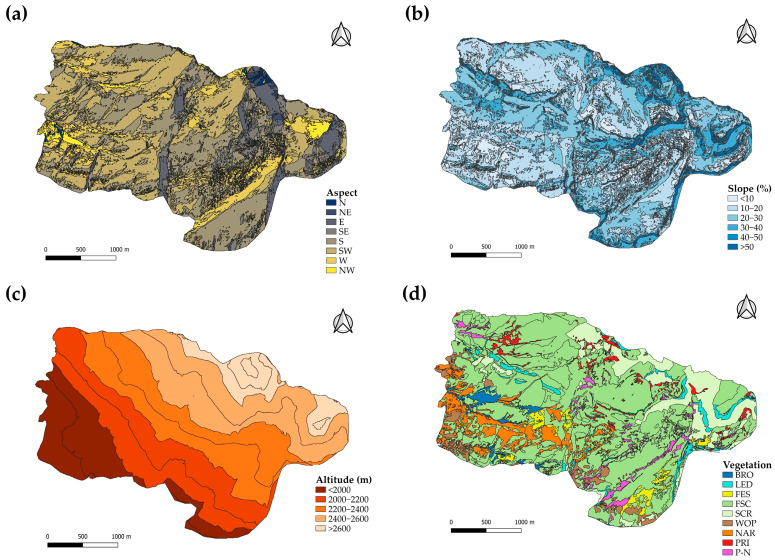
Aspect (**a**), slope (**b**), altitude (**c**), and vegetation (**d**) maps of Collarada mountain pastures (Central Pyrenees, Spain). WOP = woody plants; BRO = *Bromion erecti*; NAR = *Nardion*; FES = *Festucion eskiae*; P-N = *Primulion intricatae* and *Nardion strictae* mixtures; LED = ledge vegetation; FSC = *Festucion scopariae*; SCR = *screes*; PRI = *Primulion*.

The aspect, slope, altitude, and vegetation classes present in the study area are represented in Figure 3.

The predominant aspects are south-west- and south-facing slopes, which represent 37 and 33% of the territory, respectively, while the combination of north-, north-east-, and north-west-facing slopes covers less than 5% of the territory. Slopes of 10–20% and 20–30% are the most common in the study area, representing 70% of the territory, while flat areas of less than 10% and slopes steeper than 40% are scarce (both 7%). The majority of the territory, specifically 75%, is between 2000 and 2600 m above sea level. Regarding the vegetation classes, *Festucion scopariae* occupies most of the surface of the study area (65%), followed by screes (12%), and *Nardion* (7%). The rest of the vegetation types occupy less than 5% each.

**Figure 4:**
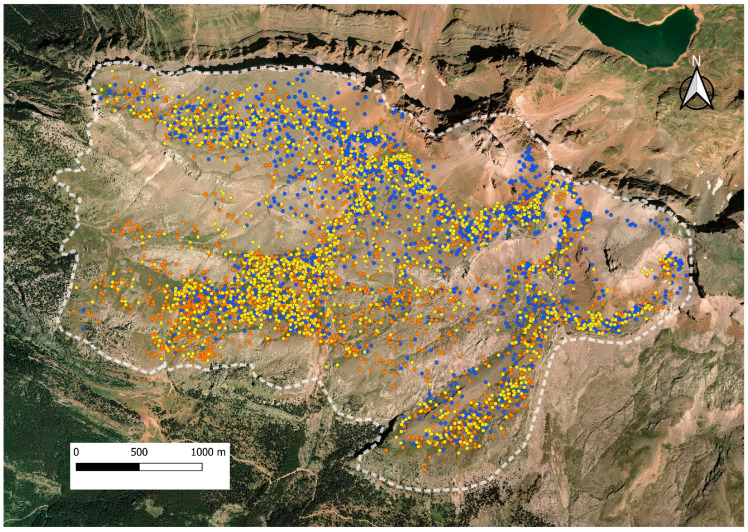
Positions of one GPS collar during grazing times (7–11 and 15–19 h) at Collarada mountain pastures (Central Pyrenees, Spain) in summers of 2019, 2020, and 2021. The positions of year 2019 are indicated in orange dots, of year 2020 in blue dots, and of year 2021 in yellow dots.

The GPS positions registered in 2019, 2020, and 2021 during the grazing periods (7–11 h and 15–19 h) are shown in Figure 4. A total of 2034, 1955, and 1835 GPS locations were used in 2019, 2020, and 2021, respectively.

**Figure 5:**
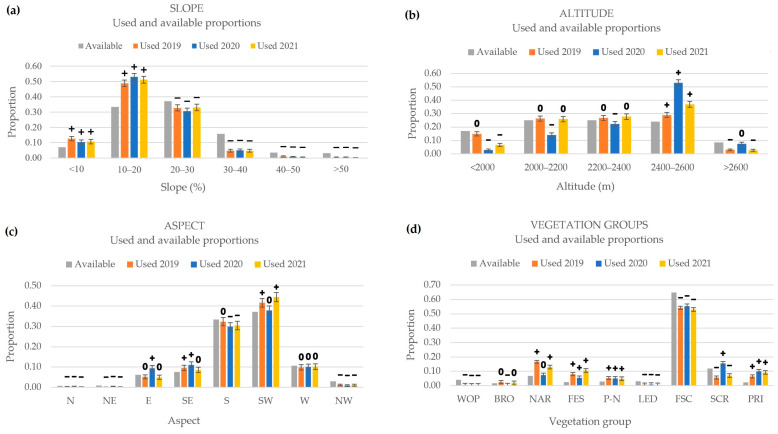
Available and used proportions of the various slope (**a**), altitude (**b**), aspect (**c**), and vegetation (**d**) categories in 2019, 2020, and 2021 at Collarada mountain pastures (Central Pyrenees, Spain). Significant resource selection was inferred after Bonferroni-corrected *p*-value. Preference is reported as “+”, “–“ means avoidance, and “0” indicates an indifferent selection. Vegetation types are ordered from lowest to highest mean altitude. WOP = woody plants; BRO = *Bromion erecti*; NAR = *Nardion strictae*; FES = *Festucion eskiae*; P-N = *Primulion intricatae* and *Nardion strictae* mixtures; LED = ledge vegetation; FSC = *Festucion scopariae*; SCR = *screes*; PRI = *Primulion intricatae*.

The available and used proportions of the various slope categories in 2019, 2020, and 2021 are presented in Figure 5a. Sheep preferred grazing slopes under 20% and avoided slopes over 20%. Figure 5b displays the available and used proportions of the various altitude categories in 2019, 2020, and 2021. The altitude range of 2400 to 2600 m was preferred for grazing. Altitudes under 2400 m were avoided in 2020, while in 2019, the flock also avoided those below 2000 m. Furthermore, sheep avoided altitudes exceeding 2600 m in both 2019 and 2020. Figure 5c represents the available and used proportions of the various aspect categories in 2019, 2020, and 2021. The south-east was selected in 2019 and 2020, the south-west in 2019 and 2021, and the east in 2020. Throughout the tree years, locations with a northern component were avoided, while sheep were indifferent to those facing west.

As shown in Figure 5d, where the available and used proportions of the different vegetation classes are represented, *Festucion eskiae*, *Primulion*, *Nardion*, and mixtures of *Primulion* and *Nardion* communities were consistently selected by the sheep throughout the three years of study. Meanwhile, woody plants, ledge vegetation, and *Festucion scopariae* were avoided by the flock. Screes were selected in 2020 but avoided in 2019 and 2021. The flock avoided *Bromion* in 2020 but showed indifference in the other two years of the study.

**Figure 6:**
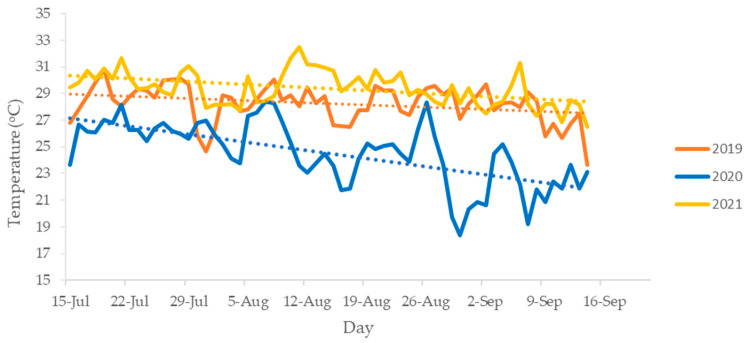
Average temperature on the surface of the sheep and trendline (dotted line) in 2019, 2020, and 2021 at Collarada mountain pastures (Central Pyrenees, Spain) throughout the summer grazing season.

The average temperature on the surface of the sheep during the study period of 3 years is illustrated in Figure 6. The mean temperature was 28.2 °C in 2019, 24.5 °C in 2020, and 29.4 °C in 2021. Differences in average temperature for the 3 years were significant (Friedman test, *p* < 0.01), with temperatures gradually descending with the advance of the summer season.

The Manly’s standardized index (Bi) for the various resource categories in 2019, 2020, and 2021 is presented in Table 1. The different categories are ranked in order of preference. No significant differences were found between the preference indexes over the 3 years (Table 1), either for aspect, slope, altitude, or vegetation group (Friedman test, *p* > 0.05). The most preferred aspect categories were SE in 2019 and 2020 and SW in 2021. Sheep preferred slopes under 10% the most in 2019 and 2021 and between 10 and 20% in 2020. The most preferred altitude was 2400 to 2600 m throughout the three years of the study. The most preferred vegetation types for the sheep were *Festucion eskiae* in 2019 and 2021 and *Primulion intrincatae* in 2019. Conversely, woody plants were consistently avoided by the sheep.

**Figure 7:**
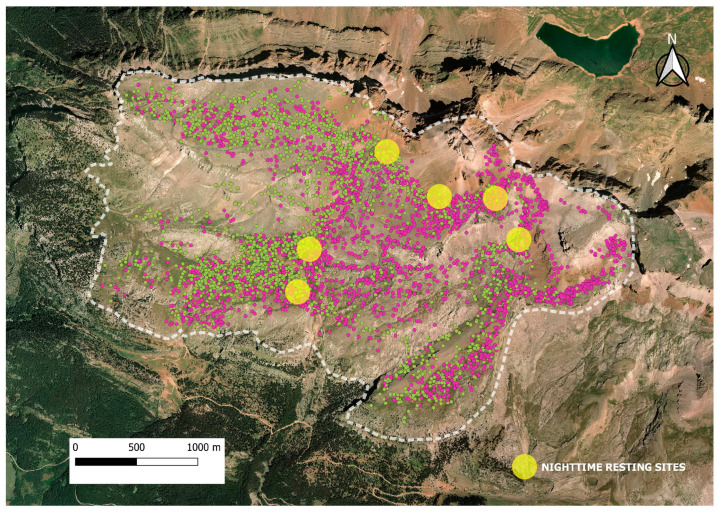
Area of study (white dash line) at Collarada mountain pastures (Central Pyrenees, Spain), overlaid by GPS positions of one GPS collar during morning (green dots) and afternoon (purple dots) grazing periods in the study period of 2019, 2020, and 2021.

The GPS positions during the morning and afternoon grazing periods in the study area for the three years of the study are shown in Figure 7. A total of 2860 GPS positions were registered in the morning grazing period (7–11 h) and 2964 GPS positions in the afternoon grazing period (15–19 h).

The results of the Manly’s standardized index for each slope, aspect, altitude, and vegetation category during the morning and afternoon grazing periods are displayed in Table 2. A statistical test shows no significant differences between the morning and afternoon grazing periods (Friedman test, *p* > 0.05) (Table 2). However, the main differences between the morning and afternoon grazing periods are in aspect, as they prefer south-west in the morning and south-east followed by east in the afternoon. In both periods, slopes under 20% are selected, while steeper slopes are avoided. The most preferred altitude range is 2400 to 2600 m, both in the morning and in the afternoon. However, the altitude range between 2000 and 2200 is preferred in the morning but avoided in the afternoon. Altitudes below 2000 m and above 2600 m are avoided during both periods. *F eskiae* is the most preferred vegetation class in the morning and *Primulion* in the afternoon hours. *Nardion* and the mixture of *Primulion* and *Nardion* are also selected by the flock; the sheep are indifferent to *Bromion* and screes in the morning and afternoon, respectively, while other vegetation classes are avoided.

**Figure 8:**
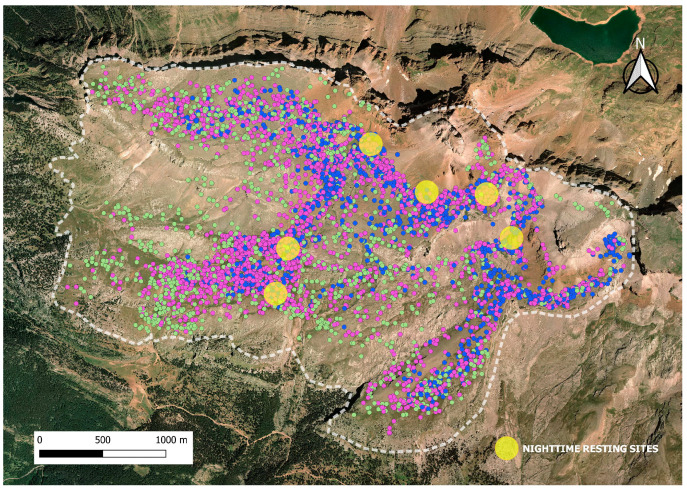
Area of study (white dash line) at Collarada mountain pastures (Central Pyrenees, Spain), overlaid by GPS positions of one GPS collar in July (blue dots), August (purple dots), and September (green dots), in the study period of 2019, 2020, and 2021.

The GPS positions for each month of the summer season in the study area for the three years of the study are shown in Figure 8. A total of 1635 GPS positions were registered from the 11 of July to the 31; 2812 positions in August; and 1377 GPS positions from the 1 of September to the 15. It was observed that the flock eats the areas closer to the nighttime resting sites first, and they progressively use more distant areas.

The results of the Manly’s standardized index for the various resource categories for each month of the summer season are presented in Table 3. At the beginning of the grazing season, in July and August, sheep prefer south-east locations, while at the end of the season, in September, they favor south-west locations. Altitudes ranging from 2400 to 2600 are preferred in July and August, but with the advance of the grazing season, the flock seeks lower altitudes. Regarding the differences in the preferred vegetation types, *Primulion* is the most preferred vegetation type at the beginning of the season, in July, and *F. eskiae* in August and September; the flock is indifferent to *Nardion* in July but selects it in the subsequent months; and *Bromion* is selected in September but not during July and August. Other vegetation classes are avoided by the flock, although screes are indifferent in July.

## 4. Discussion

Our findings show that sheep prefer south-east-, east-, and south-west-facing slopes. Pure south-facing slopes, despite representing 33% of the study area, are either treated indifferently or avoided by the flock, and slopes with a northern component are consistently avoided. The orientation of slopes, known as aspect, significantly influences soil characteristics and plant distribution. In the northern hemisphere, north-facing slopes receive less direct sunlight, leading to increased moisture retention and the growth of thicker vegetation compared to south-facing slopes, which are sunnier and prone to erosion due to sparse vegetation [33,34,35,36]. Other studies suggest that sheep tend to favor northern aspects over southern ones due to their presumably superior nutritional quality and the thermal comfort offered by this orientation [21,22,37]. However, Collarada pastures are mainly on the south-facing side of the mountain, with less than 5% of north-facing slopes found only at very high altitudes and composed mainly of rocks.

A decrease in the Manly’s preference index is noted as the slope degree increases, with the flock showing preference for slopes under 20% and avoiding greater slopes. McDaniel and Tiedeman [10] noted a decrease in sheep’s use of slopes steeper than 45% in a mountainous area in New Mexico (US). Baum [21] found that for each one-degree increase in slope, the probability of use by sheep in a mountain range declines by −0.9. Plaza [22] found that free-grazing flocks in a Spanish ‘dehesa’ ecosystem selected areas with a slope under 6%. Sheep usually avoid steep slopes when there is abundance of vegetation, but they explore steep slopes more often when vegetation is scarce [38] or when the lower parts are being grazed by cows in mixed grazing pastures [23]. Other studies have indicated that sheep generally utilize steeper slopes more often than cattle because cattle need higher grass height and a high volume of forage intake, so they graze on the lower ranges where the grass is higher and availability is greatest, and they avoid steep slopes and rocky outcrops [10,37,39]. Small ruminants, such as sheep and goats, are better suited for grazing on steep and rugged terrain in comparison to cattle [40].

Both our research and others provide evidence supporting the tendency of sheep to seek elevated terrain. Sheep graze on ridge tops and upper slopes and ascend further uphill to rest at night [10,39,41,42]. Additionally, they tend to bed down repeatedly in specific locations on elevated terrain, leading to overuse of vegetation in those areas [39,41]. Some authors suggest that the nocturnal uphill migration of sheep to higher ground is not primarily driven by nutritional requirements but rather serves to provide various benefits, including predator evasion and access to safer resting spots [41].

In our study, *F. eskiae*, *Primulion*, *Nardion*, and *Primulion–Nardion* vegetation communities were selected, and *F. scopariae* was rejected. Studies of landscape-use patterns and animal distribution have widely confirmed that domestic and native grazing herbivores select and spend more time in plant communities that offer abundant quantities of preferred forage species (reviewed by [43]). Therefore, spatial foraging decisions are strongly influenced by dietary preferences [11]. Sheep acceptability of grasses is influenced by a combination of plant structure and leaf quality attributes, with preferred species being short and non-stemmy with low dry matter, low tensile strength, and high crude protein content [44]. The grasses *Festuca eskia*, *Festuca scoparia*, and *Nardus stricta* do not have good nutritional values for sheep [45]. One might wonder why, in our work, the *F. eskiae* and *Nardus stricatae* alliances are selected by the sheep. Probably, the answer is that, as sheep are very selective in their diet, they especially consume some leguminous plants that appear in these plant alliances, such as *Trifolium alpinum*. This legume is not present in *F. scoparia* pastures, which, unlike the two previous ones, have incomplete ground cover and are rejected by livestock. On the other hand, the preference of sheep for *P. intricatae* pastures is well known due to its high nutritional value [25].

These selection patterns are repeated every year without significant differences, despite the fact that environmental conditions are not the same every year, as we have verified by analyzing the temperature on the surface of the body of the ewes. Furthermore, the results of our study show that sheep prefer locations facing south-west in the morning grazing period and south-east-facing slopes in the afternoon grazing period, while the altitude range between 2000 and 2200 m is only selected in the morning. Differences in habitat use between morning and afternoon grazing might be influenced by the location of the nighttime resting sites, often located on projecting ledges with an open view. The sheep´s attraction to these high-altitude areas is greatest toward the end of the day [42].

The differences in selection observed as the grazing season progresses might be related to the stage of development and condition of the vegetation. In the mountains, herbaceous production is staggered over time, resulting in a phenological shift as altitude increases (phenological wave). In June, peak flowering and production occur at 1600 m, while at 2400 m, snow still persists. In August, when the grass dries up at 1600 m, it is in its vegetative optimal state (both in quality and production) at 2400 m. It is estimated that this lag is about a month for every 300–500 m of elevation for the same plant species. By progressively ascending in altitude during the first half of summer and descending in the second half, herbivores can access higher-quality grass throughout the summer [23]. This study was conducted starting in mid-July, when the flock was left on its own at Collarada pastures. At this time, the snow-bed grasses in the high-altitude sectors, still short and tender, are especially attractive to sheep, while by the end of summer, the sheep start making their way down voluntarily, maybe because there is no longer much new grass available, even at the top of the mountain [42]. *Primulion*, which grows at higher altitudes, is the most preferred vegetation type at the beginning of the season (July), while *Bromion*, which we can find at lower altitudes, is selected in September but not in July and August. We also observed that the flock grazes first in areas closer to the nighttime resting sites, presumably trying to minimize the energy cost of locomotion seeking food, while more distant areas are only grazed at the end of the season, when they are forced to travel to find new grass and meet their energy requirements [46].

Other studies suggest that distance to water is a consistent primary determinant in predicting livestock grazing distribution [11,43]. This factor was not studied in this research since sheep can access several natural and artificial water sources spread over the territory and lick the dew from the pasture in the early morning.

## 5. Conclusions

The findings of our study provide valuable insights into the habitat selection of sheep in mountain pastures. We observed a clear preference for south-east-, east-, and south-west-facing slopes, while north-facing slopes were consistently avoided. We also observed a preference for altitudes between 2400 and 2600 m. Our findings also highlight the importance of slope degree, with sheep preferring slopes under 20%. Furthermore, our research identifies specific plant communities preferred by sheep, such as *Festucion eskiae*, *Primulion intricatae*, and *Nardion strictae*, while *Festucion scopariae* communities are rejected. The observed selection patterns remained consistent over the course of the three-year study, despite minor differences attributable to changes in climatic conditions. Additionally, we observed differences in habitat use between the morning and afternoon grazing periods, potentially influenced by the location of nighttime resting sites, and between the different months of the study, presumably due to changes in the stage of vegetation development as the summer season progresses. Understanding sheep preferences helps in planning grazing schedules and locations to avoid overgrazing and ensure an even distribution of grazing pressure, thereby maintaining pasture health. The insights gained can guide the placement of supplemental resources such as water and salt to align with sheep movement patterns, enhancing animal welfare and productivity. This study advances knowledge on the spatial behavior of grazing sheep, contributing to the broader field of landscape ecology and animal behavior. It underscores the importance of integrating new technologies, like GPS tracking, with traditional ecological research to uncover detailed patterns of habitat use. These findings can inform further research on the ecological impacts of grazing and the development of sustainable grazing practices in other mountainous regions.

## Figures and Tables

**Table 1:** Manly’s standardized index (B_i_) and preference rank for the different resource categories in 2019, 2020, and 2021 at Collarada mountain pastures (Central Pyrenees, Spain). Red cells and green cells indicate a significant (95% confidence interval with a Bonferroni adjustment) avoidance or preference for a specific category, respectively. Yellow cells indicate an indifferent selection. WOP = woody plants; BRO = *Bromion erecti*; NAR = *Nardion strictae*; FES = *Festucion eskiae*; P-N = *Primulion intricatae* and *Nardion strictae* mixtures; LED = ledge vegetation; FSC = *Festucion scopariae*; SCR = *screes*; PRI = *Primulion intricatae*.

	B_i_			Rank		
	2019	2020	2021	2019	2020	2021
Aspect						
N	0.05	0.06	0.02	7	6	8
NE	0.01	0.05	0.03	8	7	7
E	0.14	0.22	0.14	5	1	5
SE	0.22	0.21	0.20	1	2	2
S	0.17	0.13	0.16	3	5	4
SW	0.19	0.15	0.21	2	3	1
W	0.16	0.14	0.17	4	4	3
NW	0.06	0.04	0.06	6	8	6
Slope (%)						
<10	0.37	0.33	0.35	1	2	1
10–20	0.30	0.35	0.35	2	1	2
20–30	0.18	0.18	0.20	3	3	3
30–40	0.06	0.07	0.07	4	4	4
40–50	0.05	0.04	0.03	6	5	5
>50	0.02	0.03	0.01	5	6	6
Altitude (m)						
<2000	0.19	0.03	0.09	4	5	4
2000–2200	0.23	0.12	0.24	3	4	3
2200–2400	0.23	0.19	0.25	2	2	2
2400–2600	0.27	0.47	0.35	1	1	1
>2600	0.08	0.19	0.07	5	3	5
Vegetation						
WOP	0.01	0.00	0.00	9	9	9
BRO	0.11	0.02	0.07	5	8	5
NAR	0.18	0.09	0.13	3	5	3
FES	0.25	0.19	0.30	1	2	1
P-N	0.14	0.15	0.12	4	3	4
LED	0.02	0.02	0.01	8	7	8
FSC	0.06	0.07	0.06	6	6	6
SCR	0.03	0.11	0.04	7	4	7
PRI	0.20	0.36	0.27	2	1	2

**Table 2:** Manly’s standardized index (B_i_) and preference rank for each slope, aspect, altitude, and vegetation category during the morning and afternoon grazing periods at Collarada mountain pastures (Central Pyrenees, Spain). Red cells and green cells indicate a significant (95% confidence interval with a Bonferroni adjustment) avoidance or preference for a specific category, respectively. Yellow cells indicate an indifferent selection. WOP = woody plants; BRO = *Bromion erecti*; NAR = *Nardion strictae*; FES = *Festucion eskiae*; P-N = *Primulion intricatae* and *Nardion strictae* mixtures; LED = ledge vegetation; FSC = *Festucion scopariae*; SCR = *screes*; PRI = *Primulion intricatae*.

	B_i_		Rank	
	a.m.	p.m.	a.m.	p.m.
Aspect				
N	0.03	0.05	7	6
NE	0.01	0.04	8	7
E	0.08	0.23	5	2
SE	0.15	0.25	4	1
S	0.16	0.15	3	3
SW	0.27	0.12	1	4
W	0.22	0.11	2	5
NW	0.07	0.04	6	8
Slope (%)				
<10	0.37	0.33	1	1
10–20	0.37	0.30	2	2
20–30	0.20	0.18	3	3
30–40	0.03	0.09	4	4
40–50	0.01	0.07	6	5
>50	0.02	0.03	5	6
Altitude (m)				
<2000	0.14	0.07	4	5
2000–2200	0.25	0.14	2	4
2200–2400	0.22	0.23	3	2
2400–2600	0.31	0.41	1	1
>2600	0.07	0.15	5	3
Vegetation				
WOP	0.00	0.01	9	9
BRO	0.09	0.05	5	7
NAR	0.17	0.10	3	4
FES	0.26	0.23	1	2
P-N	0.11	0.16	4	3
LED	0.01	0.03	8	8
FSC	0.06	0.07	6	6
SCR	0.04	0.07	7	5
PRI	0.26	0.28	2	1

**Table 3:** Manly’s standardized index (B_i_) and preference rank for each slope, aspect, altitude, and vegetation category for each month of the summer season at Collarada mountain pastures (Central Pyrenees, Spain). Red cells and green cells indicate a significant (95% confidence interval with a Bonferroni adjustment) avoidance or preference for a specific category, respectively. Yellow cells indicate an indifferent selection. WOP = woody plants; BRO = *Bromion erecti*; NAR = *Nardion strictae*; FES = *Festucion eskiae*; P-N = *Primulion intricatae* and *Nardion strictae* mixtures; LED = ledge vegetation; FSC = *Festucion scopariae*; SCR = *screes*; PRI = *Primulion intricatae*.

	B_i_			Rank		
	July	August	September	July	August	September
Aspect						
N	0.07	0.04	0.02	6	8	8
NE	0.01	0.05	0.03	8	7	7
E	0.18	0.18	0.15	2	3	5
SE	0.24	0.20	0.20	1	1	2
S	0.15	0.14	0.18	3	5	3
SW	0.15	0.19	0.21	4	2	1
W	0.13	0.16	0.17	5	4	4
NW	0.07	0.05	0.04	7	6	6
Slope (%)						
<10	0.36	0.32	0.40	1	2	1
10–20	0.33	0.36	0.29	2	1	2
20–30	0.18	0.20	0.18	3	3	3
30–40	0.06	0.07	0.07	4	4	4
40–50	0.05	0.04	0.02	5	5	6
>50	0.02	0.02	0.03	6	6	5
Altitude (m)						
<2000	0.01	0.09	0.24	5	5	1
2000–2200	0.09	0.23	0.23	4	2	2
2200–2400	0.22	0.23	0.23	2	3	3
2400–2600	0.57	0.32	0.20	1	1	4
>2600	0.10	0.12	0.10	3	4	5
Vegetation						
WOP	0.00	0.00	0.01	9	9	9
BRO	0.01	0.07	0.16	8	5	3
NAR	0.06	0.17	0.15	5	3	4
FES	0.23	0.27	0.22	2	1	1
P-N	0.16	0.12	0.13	3	4	5
LED	0.01	0.02	0.02	7	8	8
FSC	0.06	0.06	0.07	6	6	6
SCR	0.08	0.05	0.05	4	7	7
PRI	0.40	0.24	0.19	1	2	2

## Data Availability

Data can be shared with other researchers upon request for collaboration.
